# Radioactive polymeric nanoparticles for biomedical application

**DOI:** 10.1080/10717544.2020.1837296

**Published:** 2020-10-29

**Authors:** Shentian Wu, Edward Helal-Neto, Ana Paula dos Santos Matos, Amir Jafari, Ján Kozempel, Yuri José de Albuquerque Silva, Carolina Serrano-Larrea, Severino Alves Junior, Eduardo Ricci-Junior, Frank Alexis, Ralph Santos-Oliveira

**Affiliations:** a Department of Radiotherapy Center, Maoming People’s Hospital, Maoming City, China; b Nuclear Engineering Institute, Brazilian Nuclear Energy Commission, Rio de Janeiro, Brazil; c Faculty of Pharmacy, Federal University of Rio de Janeiro, Rio de Janeiro, Brazil; d Department of Medical Nanotechnology in the Faculty of Advanced Technology in Medicine, Iran University of Medical Science, Tehran, Iran; e Faculty of Nuclear Sciences and Physical Engineering (FJFI), Czech Technical University in Prague (ČVUT), Prague, Czech Republic; f Department of Fundamental Chemistry, Federal University of Pernambuco, Recife, Brazil; g School of Biological Sciences and Engineering, Yachay Tech University, Urcuquí, Ecuador; h Laboratory of Radiopharmacy and Nanoradiopharmaceuticals, Zona Oeste State University, Rio de Janeiro, Brazil

**Keywords:** Nanoparticles, polymers, nanoradiopharmaceuticals, radionuclides, medical imaging

## Abstract

Nowadays, emerging radiolabeled nanosystems are revolutionizing medicine in terms of diagnostics, treatment, and theranostics. These radionuclides include polymeric nanoparticles (NPs), liposomal carriers, dendrimers, magnetic iron oxide NPs, silica NPs, carbon nanotubes, and inorganic metal-based nanoformulations. Between these nano-platforms, polymeric NPs have gained attention in the biomedical field due to their excellent properties, such as their surface to mass ratio, quantum properties, biodegradability, low toxicity, and ability to absorb and carry other molecules. In addition, NPs are capable of carrying high payloads of radionuclides which can be used for diagnostic, treatment, and theranostics depending on the radioactive material linked. The radiolabeling process of nanoparticles can be performed by direct or indirect labeling process. In both cases, the most appropriate must be selected in order to keep the targeting properties as preserved as possible. In addition, radionuclide therapy has the advantage of delivering a highly concentrated absorbed dose to the targeted tissue while sparing the surrounding healthy tissues. Said another way, radioactive polymeric NPs represent a promising prospect in the treatment and diagnostics of cardiovascular diseases such as cardiac ischemia, infectious diseases such as tuberculosis, and other type of cancer cells or tumors.

## 1. Introduction

The development of nanoscale technology is a changing science, especially in which medicine pushes disease prevention, diagnosis, and treatment frontiers ahead. These nanoparticle-based platforms have a size range from 1 to 100 nm and include functionalized carbon nanotubes, nanomachines, nanorobots, nanofibers, self-assembling polymeric nanoconstructs, nanomembranes, nano-sized chips, and metallic nanoparticles among others nanoparticles (NPs) (Singh and Lillard, 2009; Khan et al., 2019).

Nuclear medicine is a branch of medicine that uses radiation to provide functional and anatomical data from a specific organ. The modality performed in nuclear medicine is molecular imaging. This modality is the most powerful technology available for early detection of a great number of diseases, including several types of cancer, in the same way the use of beta or alpha radionuclides allows to perform molecular radiotherapy, which is the most direct and common form of radiotherapy (Zuckier, 2008; Studwell and Kotton, 2011; Kharisov et al., 2014; Mikla and Mikla, 2014; Positron Emission Tomography (PET), 2020; Nuclear Medicine and Molecular Imaging Division, 2020). The most common and well-investigated nanomaterials used in nuclear medicine include polymeric NPs, liposomal carriers, dendrimers, magnetic iron oxide NPs, silica NPs, carbon nanotubes, and inorganic metal-based nanoformulations (Michalet et al., 2005; McDevitt et al., 2007; Das et al., 2009; De Barros et al., 2012; Elsabahy and Wooley, 2012; Rybak-Smith and Townley, 2015; Ito et al., 2016; Ni et al., 2018; Farzin et al., 2019; Tang et al., 2019).

Synthetic modifications to polymers impose limitations on polymer size which are called polymeric nanoparticles. Polymeric nanoparticles include nanoparticles composed polymers such as polymer conjugates, micelles, polymersomes, etc. NPs can be prepared by several methods divided in two categories: polymerization of monomers (emulsion, interfacial or interfacial condensation) and performed polymers (single or double emulsification-solvent evaporation, emulsification-solvent diffusion, salting-out, nanoprecipitation, dialysis, and supercritical fluid) (Reis et al., 2006; Crucho and Barros, 2017). Radiolabeled polymeric NPs can be prepared by several methods: surface coupling (indirect surface labeling using a chelator, indirect surface labeling using prosthetic group, and direct surface labeling), inner incorporation (radiochemical doping, encapsulation, nonradioactive variant activation, isotope exchange, and non-isotope exchange), and interface engineering as polyethylene glycol (PEG)ylation (Ge et al., 2020).

**Figure 1.**
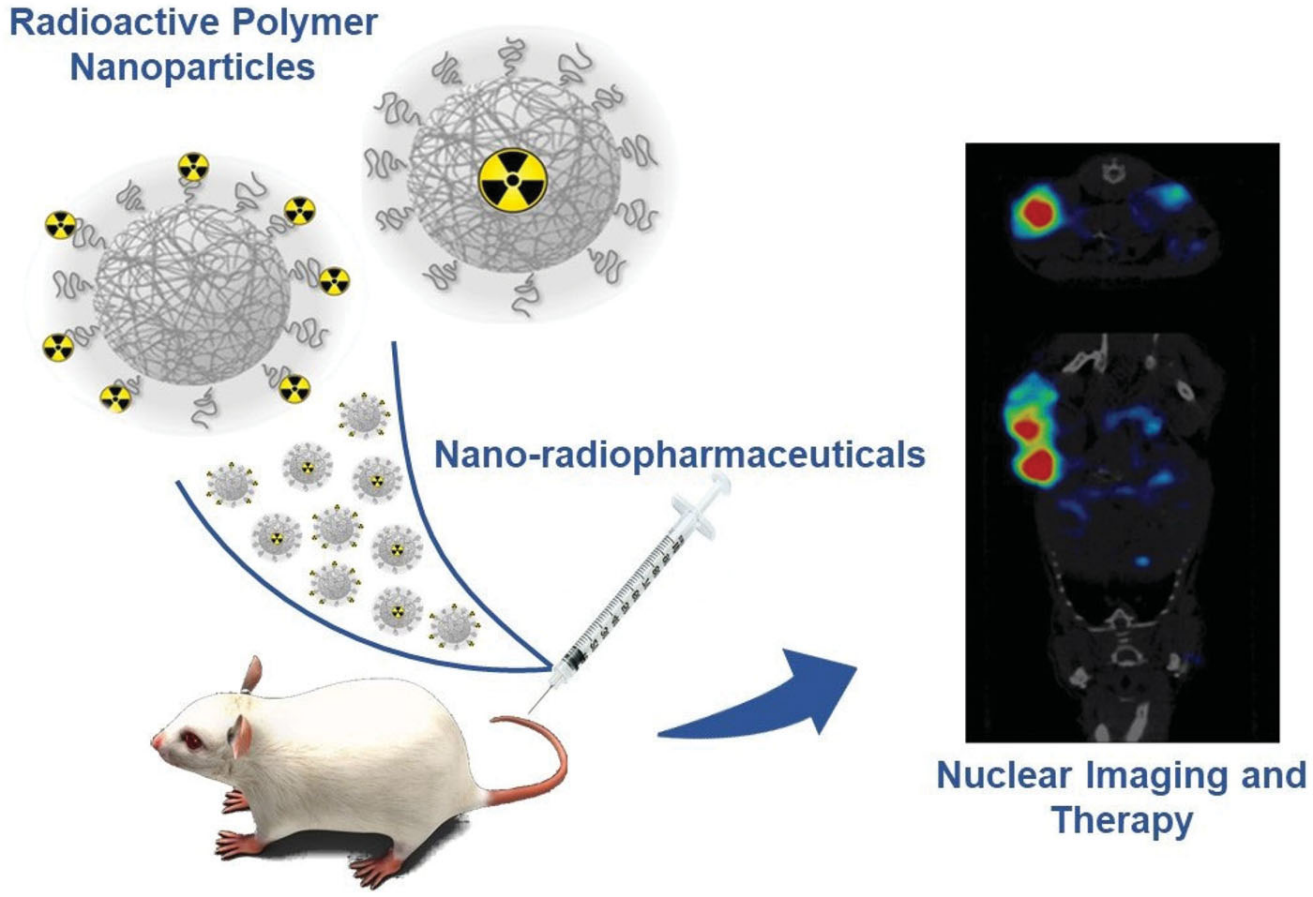
Schematic figure showing the main two types of polymeric nanoparticles and their application in imaging and/or therapy from pre-clinical data to a human use.

There are two methods to design radioactive nanosystems (Figure 1). The first method is the incorporation of a radioactive element into a nanosized cluster. For example, noble metals such as gold can be bombarded with neutrons in a nuclear reactor to generate radioactive core NPs. The second method attaches a radioactive element to a NP (nanoparticle radiolabeling). This method presents high versatility and incorporates several radioactive elements into a ligand on the NP surface. Nevertheless, to take advantage of these properties, there are some requirements to avoid recognition by the mononuclear phagocyte system (MPS) and concomitant uptake by the reticuloendothelial system (RES), followed by relatively long blood circulation times (Kolishetti et al., 2011; Morales-Avila et al., 2012; Lewis and Kannan, 2014; Srivatsan and Chen, 2014).

This review aims to provide an overview of polymeric nanoparticles labeled with radionuclides for biomedical applications such as therapy and diagnosis.

## 2. Polymeric nanoparticles

Polymers are large molecules composed of many repeated subunits (Ista et al., 1999) and these macromolecules have been successfully applied in physics, chemistry, biology, and interdisciplinary studies like biomedicine. Polymers have undeniable advantages toward inorganic materials in terms of cost, unique physical properties (e.g. flexibility based on functionalization), diversity, and ease of accessibility to building-block sources. In short, polymers have a different synthesis method: evaporation, nanoprecipitation (solvent displacement), salting-out, dialysis, supercritical fluid (SCF) technology, emulsion, interfacial polymerization, and controlled living radical polymerization. Polymers adapt different forms like block copolymer micelles, polymer conjugates of proteins, polymeric drugs, aptamers, and combination of nonviral vectors through covalent linkages. The most prominent types of conjugated polymers are polyaniline, polypyrrole, polyacetylene and its derivatives which have been intensively studied due to their intrinsic conductivity, polythiophenes, polyphenylenes, polyfluorenes, poly(arylenevinylene), and poly(phenyleneethynylene). These types are of great interest due to their electro-optical and photoluminescence properties (Pecher and Mecking, 2010).

Several other factors influence polymers, namely, degradation rate, including polymer chemical structure, composition, physicochemical factors (ionic charge, ionic strength, and pH), physical factors (shape and size), morphological aspect (amorphous, semi-crystalline, crystalline, microstructure), degradation mechanism (enzymatic, hydrolysis, microbial), molecular weight, and route of administration (i.e. intravenous, subcutaneous, among others) (Pillai and Panchagnula, 2001; Alexis, 2005). By using in vivo application, based on omnipresent toxicological literatures, biodegradable nanoparticles have an advantage over non-biodegradable nanoparticles because they are fully absorbed or eliminated from the body and usually require no further treatments for removal of possible accumulation inside the body (Yasukawa et al., 2004).

Polymeric NPs are colloidal particles solid in nature, which possess unique features such as higher surface to mass ratio, quantum properties, biodegradability, lower toxicity, and the ability to adsorb and carry other molecules (Schmidt and Malwitz, 2003; Alexis et al., 2008; Pecher and Mecking, 2010; Lu et al., 2011). Polymeric NP application in the drug delivery field is one of the most important issues in pharmaceuticals.

Depending on their synthesis, polymeric NPs can form two types of structures used in drug delivery: nanosphere (matrix where the drug is uniformly dispersed) and nanocapsule (the drug is embedded in a cavity and the cavity is surrounded by a polymeric membrane) (Sharma, 2019). The development of polymeric NPs requires that the major component is comoposed of a polymer and includes drug/polymer conjugates, polymer micelles, polymersomes, and nanoparticles (Fonseca et al., 2015). Two important features must be taken into account when developing polymeric NPs as drug delivery systems: (1) the chemical characteristics of the polymer should not compromise the action of the active ingredients and (2) the physical properties of the polymer must be consistent and reproducible (Villanova et al., 2010). Although the definition of NPs describes their dimensions between 1 and 100 nm, in the area of drug delivery, relatively large (size > 100 nm) NPs may be needed to load a sufficient amount of drug onto particles (Baran et al., 2002; Cascone et al., 2002; Kipp, 2004).

## 3. Radionuclides

Physical radiation from radioactive species is responsible for the radioactive polymeric NP emission with beta (β) or alpha (α) emitters for therapy purposes while polymeric NPs with gamma (γ) or positron emitters are used for diagnostic targets (Müller et al., 1996; Hamoudeh et al., 2008; De Barros et al., 2012). The ideal radioactive polymeric NP should be able to target tissues and restrict radiation from spreading to other healthy tissue around the target. In addition, radioactive polymeric NPs should remain in the body for a short period of time so as to avoid prolonged patient exposure to radiation, but long enough to allow the acquisition and processing of images via computers and as well as release of therapeutic active agents (Jahangirian et al., 2017).

### 3.1. Radionuclides for therapy

Therapeutic agents containing radioactive species are based on the administration route of radioactive substances (orally or parenterally), which will be concentrated in an organ or site for sufficient time to deliver a therapeutic dose of radiation. In order to achieve the therapeutic effect, only β and radioactive α species can be used because they allow very high ionization per length of travel (Müller et al., 1996; Kipp, 2004; Hamoudeh et al., 2008; Jahangirian et al., 2017) (Table 1). Radionuclide therapy has the advantage of delivering a highly concentrated absorbed dose to the targeted tissues while sparing the surrounding healthy tissues (De Barros et al., 2012).

**Table 1.** Main radionuclides used in therapy and their main properties. IT: isomeric transition; EC: electron capture.

Radionuclides	Half-Life	Radiation
^177^Lu	6.73 days	β: 0.490 MeV γ and X-ray: 0.113 MeV (3%),0.210 MeV (11%)
^153^Sm	1.93 days	β: 0.810 MeV (20%), 0.710 MeV (30%), 0.640 MeV (50%) and γ photons of 103 keV (28%)
^131^I	8.02 days	β: 0.607 MeV (89.6%), 0.334 MeV (7.23%) and γ photons of 0.364 MeV (81.5%), 0.284 MeV (6.12%) 0.637 MeV (7.14%)
^32 ^P	14.26 days	β: 1.71 MeV (100%)
^89^Sr	50.53 days	β: 1.501 MeV (99.99%)
^90^Y	64.10 h	β: 2.280 MeV (99.98%)
^117m^Sn	13.60 days	IT: γ photons 0.158 MeV (86.4%) and 0.156 MeV (2.11%)
^169^Er	9.40 days	β: 0.351 MeV (55%) and 0.342 MeV (45%)
^186^Re	3.72 days	β: 1.071 MeV (70.99%), 0.934 MeV (21.54%) and γ photon of 0.137 MeV (9.47%) EC: X-ray of 50.32 KeV (3%)
^188^Re	17.00 h	β: 2.12 MeV (70.7%), 1.965 MeV (25.8%) γ photon of 0.155 MeV (15.49%)
^223^Ra	11.44 days	α: 5.71 MeV (51.6%), 5.606 MeV (25.2%), 5.539 MeV (9%) and 5.747 MeV (9%)

The ideal radionuclide (radioactive species) chosen for therapy relies on two main factors. The first comprises physical factors like type of emission, energy radiation, daughter product, method of production, radionuclide purity, and effective half-life (a relation between physical half-life and biological half-life), which influence medical internal radiation dosimetry (MIRD) and linear energy transfer (LET); for therapeutic radionuclides it should be very high. The second includes biochemical factors such as tissue targeting, retention in tumor, stability, and toxicity. In some β-emitting radionuclides it is possible to observe secondary γ-radiation decay. In this case, these radionuclides allow therapy but also diagnosing and completing the full terminology of theranostics (Yeong et al., 2014). Moreover, radionuclides that emit energetic α- or β-particles are most commonly used to treat dense and large tumors; otherwise, for treatment of small clusters of cancer cells or small tumors, Äuger electrons emitting radionuclides are preferred due to their high-level cytotoxicity and short-range biological effectiveness (Hong et al., 2009; Urakami et al., 2009; Ersahin et al., 2011; Yeong et al., 2014; Koziorowski et al., 2017; Bavelaar et al., 2018).

### 3.2. Radionuclides for imaging

The use of radionuclides for imaging (Table 2) is a unique technique which provides molecular and sub-molecular imaging of a live subject (Hong et al., 2009; Urakami et al., 2009; Ting et al., 2010; Ersahin et al., 2011; Uhl et al., 2015; Koziorowski et al., 2017). In this way, choosing the appropriate radionuclide is a crucial step in designing efficient radionuclides by considering the targeted and normative manner which relies on physical half-life, decay mode, and emission properties. Thus, for imaging purposes, γ emitter’s radionuclides with energy between 130–370 keV are recommended for SPECT (Single Photon Emission Computed Tomography) (Jadvar et al., 2018; Waaijer et al., 2018). However, in the case of high-energy positron emitter (511 keV) descendants from the annihilation process, they can be applied to PET (Positron Emission Tomography) (Altai et al., 2017; Mir et al., 2017; Qi et al., 2017; Lamb and Holland, 2018).

**Table 2.** Main radionuclides used in imaging and their main properties.

Radionuclide	Production	Emission Type	Half-Life	Emax(γ) (keV)
^131^I	^130^I(n, γ)^131^Te (β) ^131^I	γ (81.2%), β	8.0 days	284, 364, 637
^67^Ga	^68^Zn (n, p)^67^Ga	Γ	78.3 h	93, 184, 300, 393
^111^In	^111^Cd (p, n)^111^In	Auger, γ	67.2 h	171, 245
^123^I	^121^Sn (α, 2n)^123^I	Auger, γ	13.2 h	159
99mTc	^99^Mo/9 9 mT c-generator	Γ	6.0 h	140
^18^F	^18^O (p, n)^18^F	Positron	1.83 h	Eβ + 635
^64^Cu	^64^Ni(p, n)^64^Cu	Positron	12.7 h	Eβ + 656
^76^Br	^76^Se(p, n)^76^Br	Positron	16.0 h	Eβ + 3941
^124^I	^124^Te(p, n)^124^I	Positron	100.2 h	Eβ + 2134, 1533

The first radiopharmaceutical was iodine 131 (^131^I) which was used for thyroid gland imaging. This commonly used radioisotope uses its γ emission for imaging and its β emission for therapeutic purposes. However, the release of ^131^I and ^131^I-tyrosine in the blood represents a potential health risk (Schuster et al., 2016). In this context, Fard-Esfahani et al. reviewed the adverse effects of iodine-131 which comprised “early complications” including dry eye, radiation thyroiditis, gastrointestinal symptoms, nasolacrimal duct obstruction, sialadenitis/xerostomia, bone marrow suppression, and gonadal damage. Moreover, the “late complications” comprised pulmonary fibrosis, secondary cancers, and permanent bone marrow suppression (Fard-Esfahani et al., 2014).

## 4. Radioactive polymeric nanoparticles for imaging and therapy

**Figure 2.**
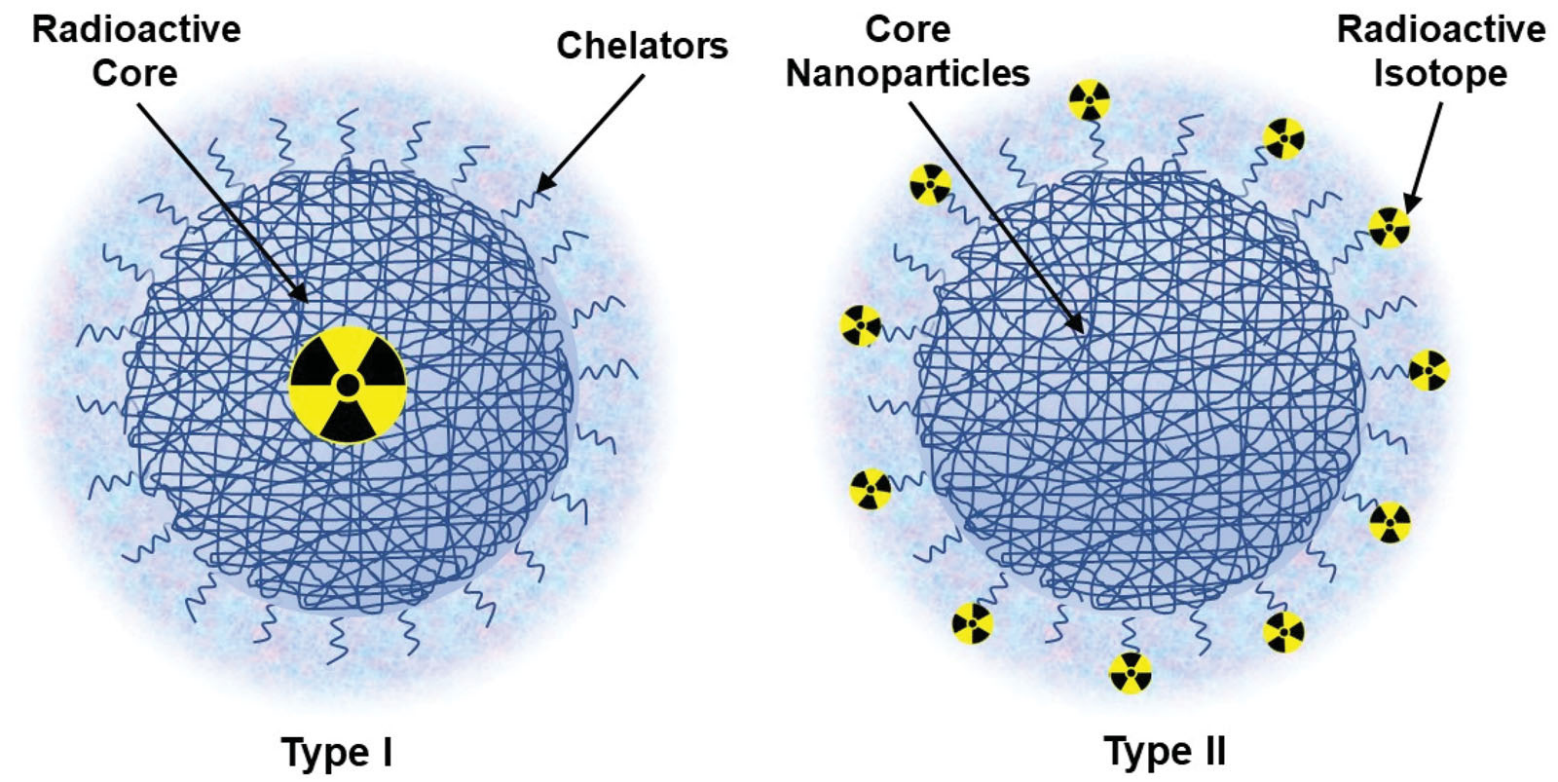
Schematic of radioactive polymer nanoparticles (NPs). In the Type I configuration, the radioactive elements are incorporated into a nano-sized cluster, whereas in the Type II configuration, the radioactive elements are decorated onto the NP surface.

Radioactive NPs provide not only functional and molecular images (Table 3) but are also useful for diagnosis by taking into account that therapeutic applications of theranostic nanoparticles have raised expectations. For imaging objectives, radioactive polymeric NPs can be designed by two possible methods (Figure 2).

**Table 3.** Some of the nanoparticles used for nuclear imaging.

Modality	Image Probe (Amount of Probe)	Type of Radiation	Sensitivity	Spatial Resolution	Tissue Depth	Nanoparticle Design
SPECT	99 mT c, 111 In, etc., loaded or labeled nanoparticles (ng)	γ-ray	10 − 10-10 − 11 (pM)	0.5–1 mm	No limit	Surface bio-conjugation or after loading
PET	18 F, 64 Cu loaded or labeled nanoparticles (ng)	Positron high energy γ-ray	10 − 11-10 − 12 (pM)	1–2 mm	No limit	Surface bio-conjugation or after loading

The first method involves the incorporation of a radioactive element in a nanosized cluster. Despite the advantages of the omnipresent method, complications like oxidization of radioactive elements or eluding the nanoscale imaging remain a challenge. Noble metals such as gold can be bombarded with neutrons in a nuclear reactor to generate radioactive core NPs. The second method involves attaching a radioactive element to a NP (also called radiolabeling of a particle). This method is versatile and can incorporate various radioelements of choice into a ligand on the NP surface using functionalization chemistry. Subsequently, these bifunctional chelators are used to carry metallic radionuclides. However, the dissociation of the radionuclide under in vivo conditions could result in false or disorientated images (Schuster et al., 2016).

Among the considerable number of developed NPs pre-clinically and clinically tested, radioactive NPs based on polymeric NPs possess great advantages. When compared to the usual radionuclides treatment, radioactive NPs are capable of carrying high payloads of radionuclides for noninvasive imaging and/or therapy. They can be used for nuclear imaging or radiotherapy depending on the type of radioactive material used in the final composition (El-Say and El-Sawy, 2017).

Radioactive NPs have the potential to significantly improve the medical outcomes of several therapies and diagnostics by enhancing the accumulation of the drug embedded into diseased tissue target sites through passive or active targeting. They can also be used for theranostics. In delivery systems undertaking passive targeting, polymeric NPs accumulate into pathological sites with leaky vasculatures (through 100–1000 nm gap size) of tumor due to the enhanced permeability and retention (EPR) effect. The EPR effect has two aspects: In brief, the first belongs to “enhanced’’ permeation arising from ‘‘leaky’’ vasculature of a tumor; large gaps are created during an angiogenesis procedure (or even de novo vascularization) and these gaps’ tasks are to nurture tumor cells. In this way, an ideal escape route provided during polymeric blood circulation accumulates in the tumor site. The second aspect, in solid tumors, refers to the ‘‘retention’’ effect where lymphatic drainage is unable to properly drain, or transport trapped or extravasated macromolecules in normal tissue into blood circulation. The key elements in this process (i.e. EPR effect) are the polymer surface characteristics and polymeric particle size. They imply that, in terms of passive targeting, polymeric nanosized systems need to be large enough to limit the amount of extravasation and can be found through continuous capillaries; as a result, distribution throughout the body is minimized. Comparing the abnormal cells capillary system with normal ones provides a good range of polymeric particle size in which the most relied range was about >100 nm (Hong et al., 2009; Fard-Esfahani et al., 2014; Koziorowski et al., 2017; Chen et al., 2019). In contrast, active targeting is achieved by surface decoration or presence of moiety on NPs with targeting ligands that bind to overexpressed receptors on the diseased tissues. Active targeting features can also be incorporated into the nanostructures by including stimuli-responsive components into the nanomaterials. Ideally, both targeting mechanisms aim to concentrate the nanomaterials while containing the embedded drugs and/or diagnostic probes in diseased tissues, avoiding drug accumulation or drug-release at healthy tissues (Du et al., 2014 Jul-Aug; Shukla et al., 2016).

In this way, several methods have been developed to radiolabel polymeric NPs using radiometals and radiohalogens. Numerous radionuclides have been used to prepare radioactive polymeric NPs. As described by Psimadas et al., ^111^In-labeled NPs have been widely used to understand the biodistribution of NPs and it was reported that diethylenetriaminepentaacetic acid (DTPA)-derivatized liposomes and micelles radiolabeled ^111^In and ^177^Lu. High radioactivity concentration in healthy Lewis rats demonstrated accumulation of particles in the liver and spleen without metals releasing from the complexes; moreover, they did not show high intestine excretion 12 h after injection (Psimadas et al., 2012; Lim et al., 2016). In addition, ^111^In-labeled gold nanoparticles have been reported to target αvß3 integrin in vitro and in vivo using human melanoma and glioblastoma model*s*. In another study, ^111^In-labeled polymeric nanoparticles incorporating a ruthenium-based radiosensitizer were reported to achieve combinational and targeted therapeutic effects in cancer cells that overexpress EGFR (Human Epidermal Growth Factor Receptor) (Ng et al., 2014; Gill et al., 2018).

^64^Cu decay occurs by three processes: positron, electron capture, and β decay. In addition, this radiometal is one of the most studied copper isotopes due to its potential in imaging and therapy applications. For instance, ^64^Cu has a long half-life and achieves sufficient uptake through this considerable contrast. Furthermore, in terms of coordinating chelators, copper is very poor for antibodies, proteins, peptides, and other small molecules in linking copper-based radiolabeling systems in biodistribution studies; it was reported that these systems tend to accumulate in the liver, intestine, and kidney (Zhou et al., 2019).

^99m^Tc stands out for being used in 90% of diagnostic procedures in nuclear medicine (Costa et al., 2019). Nano radiopharmaceuticals based on ^99m^Tc and, more recently, rhenium-186 have become essential tools for the diagnosis and therapy of various diseases or dysfunctions of organs and systems within the human body (Dewanjee, 1990; Hua et al., 2005; Costa et al., 2019). The development of nano radiopharmaceuticals provides a new paradigm for nuclear medicine and radioprotection and dosimetry and emerges as a viable alternative to tumor treatment and diagnosis (Garnett and Kallinteri, 2006). In addition, other radiopolymers including strontium-89 chloride, samarium-153 lexidronam, and rhenium-186 etidronate, are currently used in the treatment of bone pain caused by bone metastasis. Although there are several differences between these radiopharmaceuticals, including physical half-life, beta energy, penetration range, and biochemical features, there is no reported advantage in the increased response rate (Paes and Serafini, 2010). Technetium-99m is the most widely used SPECT radionuclide because it has optimal imaging characteristics, including a short half-life of 6.0 h and a γ emission of 140 keV for SPECT imaging applications. NPs have been labeled with ^99m^Tc to increase understanding of their biodistribution characteristics. Radiolabeling with ^99m^Tc is usually accomplished using two different methods (Sogbein et al., 2014). In the case of polymeric NPs, radiolabeling was performed through a direct labeling approach and a nicotinic acid (HYNIC)-type ligand system was used for labeling with ^99m^Tc (Kovacs et al., 2014).

Polymeric radioactive NPs can be obtained by direct irradiation of NPs, direct labeling process, using radioactive species as raw materials, or indirect labeling process using radioactive species as raw materials (Lamb and Holland, 2018). Direct irradiation can be performed with neutrons (generally in nuclear reactors) or with ion beams in particle accelerators (cyclotrons). The main concern about the direct irradiation of NPs, especially polymeric NPs, lies in the nanostructure damage caused by the high γ-radiation background as well as by the heating caused during the irradiation process (Haume et al., 2016; Lamb and Holland, 2018). The direct radiolabeling process is an easy and rapid way to produce NPs. In this process, the introduction of the radioactive material occurs without the use of chelating agents, and four main methods are used to perform the radiolabeling (Sugiura et al., 2014; Licia et al., 2017). First, radiochemical doping comprises all the processes in which radiolabeled polymeric NPs are obtained by the addition of small amounts of a radionuclide species during NP fabrication. This methodology is based on the radioactive coprecipitation governed by the Fajans–Paneth–Hahn law. According to the Fajans–Paneth–Hahn law, a radioactive trace element coprecipitates in the presence of a larger amount of carrier material. Thus, controlling experimental conditions (solubility, precipitants concentration, ionic strength, and counter ion identity) is possible to generate so-called mixed precipitates in which the radionuclide is incorporated into the nanoparticulate structure. In addition, according to the Fajans–Paneth–Hahn law, when NPs acquire a surface charge opposite the charge from the radioactive element, coprecipitation of the radioactivity element occurs (strongly dependent on the conditions used) with its chemical or physical absorption onto the NP’s surface. Regarding polymeric NPs, the presence of monomers helps chemical and physical adsorption (De Freitas et al., 2018). Secondly, physisorption is based on the surface chemistry of polymeric NPs in which small molecules or ions interact and associate with a molecular surface by electrostatic attraction or van der Waals interactions. In this case, polymeric NPs dispersed as a colloidal solution, usually acquire surface charge (electric double-layer potential). These “charged” NPs, when in contact with radioactive ions (with an opposite charge from the NP), can immobilize on the stationary layer between the particle surface and the dispersed medium. In this type of reaction, no discrete covalent or dative covalent bond is observed (Oda et al., 2017; Shi et al., 2017; Liang et al., 2018). Thirdly, direct chemisorption of polymeric NPs is direct and chemically bonded with the radionuclide. In this process, radioactive materials utilize the hydroxyl, methyl, and carbonyl groups present in most polymers and react directly with the surface polymer of polymeric NPs. In the case of ^99m^Tc-polymeric NPs, using the chemical coordination from the ^99m^Tc, a very stable octahedral geometry is made, providing in vivo stability (Dewanjee, 1990; Cheng et al., 2017; Chakravarty et al., 2017; Ni et al., 2018; Hajiramezanali et al., 2019; Sun et al., 2019). It is important to observe that chelate-free methodologies showed no disruptive effect on the physical and biochemical properties of the polymeric NP. Lastly, in cavity encapsulation, the radioactive species is trapped in the polymeric NP by a physical encapsulation process. In this case, radioactive material is encapsulated during the production of the polymeric NPs (Sun et al., 2019).

**Figure 3.**
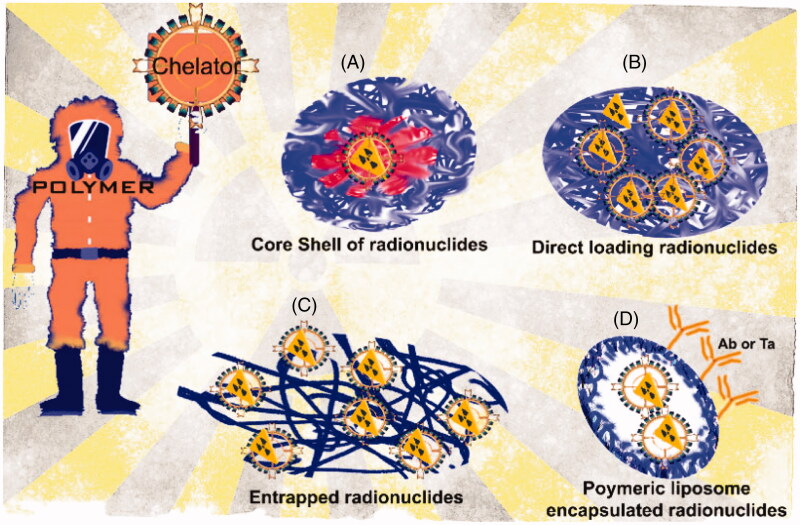
Schematic variations of radioactive polymeric nanoparticles. (A) Entrapment of the radionuclide in the core of the polymeric nanoparticle core using a chelator to increase the affinity. (B) Physisorption of radionuclide with polymeric nanoparticle. In this case the use of chelators is avoided. (C) Chemisorption of radionuclides with the use of chelators in order to conjugate with a previously entrapped compound (i.e. proteins, peptides, etc.). (D) Entrapment of radionuclides into polymeric liposomes trapped in the lipid bilayer without alteration in the membrane structure, making possible the decoration with monoclonal antibodies, for instance.

**Figure 4.**
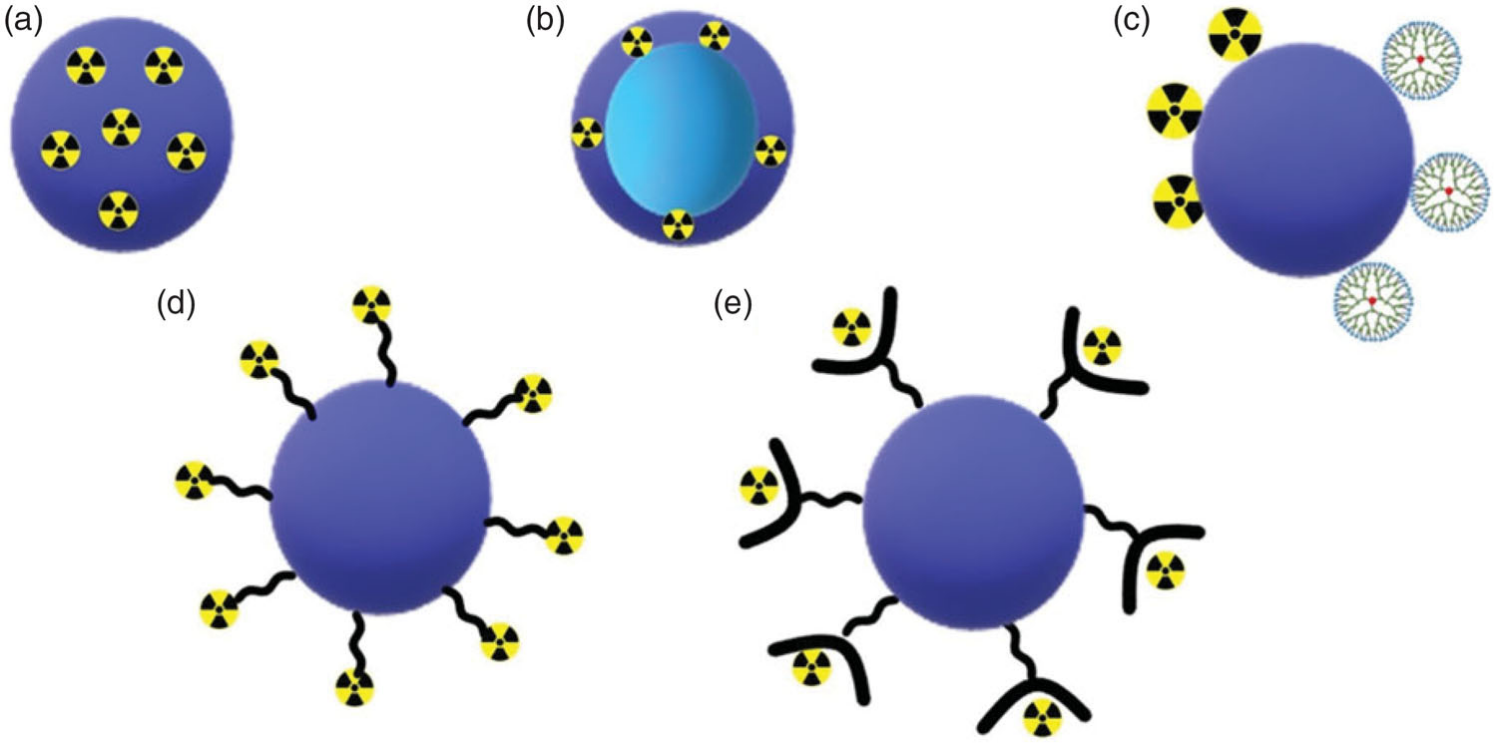
Different types of polymeric nanoparticles design for therapeutic and diagnostic applications. (a) Nanosphere with radioactive material loaded into polymeric matrix; (b) nanocapsule containing radioactive material in the polymeric shell; (c) radioactive material and dendrimers attached to polymeric nanoparticles; (d) surface modification with radioactive material attached to polymeric NP by direct labeling process; and (e) surface modification with radioactive material attached to polymeric nanoparticles by indirect labeling process.

In the indirect radiolabeling of polymeric NPs with radioactive species, two principal methodologies based on the chelating agents are used: surface-modified NPs and coated modified NPs. The surface modified nanoparticle methodology is used when polymeric NPs are coated or decorated with reactive surface groups. To achieve a highly stable complex, reactive surface groups should be biochemically recognizable. Thus, prosthetic groups are excellent options. Among a large number of reactive surface groups, organic ones as vitamins and sugar and inorganic ones as metal ions are the best (Table 4). In this model, reactive surface groups allow covalent attachment of radionuclides providing thermodynamic, kinetically, and metabolically stable mainstays ensuring that the radionuclide remains associated with the polymeric NP in vivo. The problem with this methodology is multiple radiolabeling steps with limited radiochemical yields. The disadvantage of introducing a prosthetic group (the non-protein acid constituent of conjugated protein) or metal ion chelate is handling a highly toxic NP (Ni et al., 2018; Hajiramezanali et al., 2019; Sun et al., 2019). The coated modified nanoparticle method uses functional groups on the surface of polymeric NPs, where it is possible to use chelators as hydrazinonicotinamide (HYNIC) to bind radioactive species to the NP’s surface. These chelators use the chelate-based chemistries associated with the radiochemistry from the radioactive species in order to acquire a radioactive NP; most of the recent researches strategies and methods are listed in Figures 3 and 4. Although chemistry seems to be very easy and rapid, the high cost of the chelators may be a limitation (Shaffer et al., 2016; Farrag et al., 2017; Mirkovic et al., 2019).

**Table 4.** General information about utilized polymeric nanoparticles for nuclear imaging.

Polymer/Chelate	Smart/Responsive/Specific Targeting Moieties	Radio-Metal	Labeling Method	NP Production Method	Modality	Reference
poly(lactic-co-glycolic acid) linked to polyethylene glycol	–	^99m^Tc	hydrazinonicotinamide and hydrazinobenzoic acid (co-ligand systems)	solid phase peptide synthesis	Gama scintigraphy	Franchini et al. (2012)
polyethylene glycol (PEG) (liposomal form)	arginine-glycine-aspartic acid (RGD) peptide targeting specific sequence	^125^I and ^111^In	direct method	solid phase peptide synthesis method	SPECT/CT	Rangger et al. (2012)
glycol chitosan NPs	–	^64^Cu	click chemistry via azide − alkyne cycloaddition strategy and (DOTA (dodecane tetraacetic acid)) chelator	simple solid phase synthesis	microPET	Lee et al. (2013)
poly(lactide(co-glycolide)) (PLGA)	in situ loading of mebrofenin inside PLGA	^99m^Tc	radiometal − chelator complexes	emulsion solvent evaporation	Gama scintigraphy	Subramanian et al. (2013)
polyethylene glycol (PEG)-modified gold nanorod	–	^131^I	direct: chemisorption of elemental iodine onto the GNRs (Gold nanorods) surface	seed-mediated growth method for gold nanorod	Gamma imaging	Eskandari et al. (2013)
shell by using acrylic acid (AA) and methyl methacrylate (MMA) and divinyl benzene (DVB) as cross-linker	pH-responsive with loaded doxorubicin	^99m^Tc	direct method	template method for magnetic hollow micro-spheres; free radical emulsion polymerization for polymer	Gama scintigraphy	Efthimiadou et al. (2014)
poly(lactic-co-glycolic acid) with polyethylene glycol: co-polymer PLGA-b-PEG	magnetically ‘driving’ the NP system via magnetic NP	^99m^Tc	hybrid core–shell nanosystems (organic coating)	nanoprecipitation technique	Gama scintigraphy and SPECT/PET	Psimadas et al. (2014)
polyethylene glycol	arginine-glycine-aspartic acid (RGD) peptide	^111^In	radiometal − chelator complexes	lipid film hydration	micro-SPECT/CT	Rangger et al. (2013)
avidin-modified substrates of poly(lactic-co-glycolic acid) (PLGA)	–	[^18^F]-fluorobenzylamide-poly (ethylene glycol)4-biotin	biotinylated radioligand (non-covalent)	single emulsion and size-fractionated via sequential centrifugation	PET	Sirianni et al. (2014)
1,2-distearoyl-sn-glycero-3-phosphoethanolamine-N-(methoxy (polyethylene glycol)-2000) functionalized with diethylenetriaminepentaacetic acid (DTPA)	–	^99m^Tc	radiometal − chelator complexes	solvent evaporation method	Gamma scintigraphy	Shi et al. (2017)
modified PEGylation on NP surface	containing: MoS_2_ nanosheet, iron oxide NP	^64^Cu	chelator-free manner	Morrison method for MoS_2_ and classical thermo-decomposition for iron oxide	PET	Liu et al. (2015)
polyester based NPs	targeting peptide AGBBB015F (15 F)	4-[^18^F] fluorobenzyl-2 bromoacetamide= [^18^F]FBBA	three strategies employed: core shell, entrapped, and direct loading	co-precipitation	PET-CT	Di Mauro et al. (2015)
poly(lactic-co-glycolic acid)	antifungal drug voriconazole	^99m^Tc	direct loading	emulsion solvent evaporation	Gamma scintigraphy	Das et al. (2015)
poly-D,L-lactic-co-glycolic acid based NP	ursolic acid (drug)	^99m^Tc	direct loading	emulsion solvent evaporation	Gamma scintigraphy	Baishya et al. (2016)
chitosan grafted with poly-D-lactide (PDLA-C); PEG-modified (PEG-PDLA-CS); monomethoxy(polyethylene glycol)- poly(lactic-co-glycolic acid)-polylysine; PEG-modified monomethoxy (polyethylene glycol) phosphatidylserine/calcium phosphate hybrids).		N-succinimidyl 4-[^18^F] fluorobenzoate	direct loading	sonicating; double emulsion method; biomineralization	microPET	Wang et al. (2015)
carbon NP with an encapsulated core of ^99m^Tc was coated with the polycation protamine sulfate and attached to anionic polystyrene sulfonate microspheres	–	^99m^Tc-labeled macroaggregated albumin	core shell	Technegas™ and Browitt sonicating precipitator	SPECT/CT	Stephens et al. (2019)
poly(4-vinylphenol)	rat anti-mouse CD31/ PECAM-1 mAb; rat anti-mouse ^TM^ mAb;	^124^I	direct conjugation	solvent diffusion method	PET	Simone et al. (2012)
poly(ethylene glycol)-block-poly (g-benzyl-L-glutamate) (PEG-b-PBLG) micelles and poly(trimethylene carbonate)-block-poly(glutamic acid)	–	^99m^Tc	direct loading	nanoprecipitation method (solvent-assisted dispersion)	Gamma scintigraphy	Psimadas et al. (2014)
surface-PEGylated of high generation of dendrimers	–	^99m^Tc	radiometal − chelator complexes	click reaction	SPECT	McNelles et al. (2015)
polyethylene glycol-based liposome	–	^68^Ga^3+^	specific chelator (DSPE-PEG (1,2-distearoyl- sn-glycero-3- phosphoethanolamine-n- [(carboxy (polyethyleneglycol )2000](ammonium salt) )-NODAGA) (1,4,7-triazacyclononane,1-glutaric acid-4,7-acetic acid )	water-in-oil emulsion	PET/MR (Magnetic Ressonance)	Malinge et al. (2017)
poly(2-ethyl-2-oxazoline) and poly(N-(2-hydroxypropyl) methacrylamide) (PHPMA)	–	^99m^Tc-HEDP	direct loading	adsorbed layer on hydroxyapatite	SPECT/CT	Lobaz et al. (2019)
polyethylene glycol–polylactic acid	resveratrol (RSV) with anti-tumor activity	^18^F- fluorodexoy-glucose	direct loading	lyophilization	PET/CT	Jung et al. (2015)
poly (L-lactic acid)	–	^188^Re	direct loading	radiomicrospheres by solvent evaporation method	–	Jamre et al. (2018)
N,N,N-trimethyl chitosan (TMC)-coated magnetic nanoparticles with (DOTA) as a radioisotope chelator	bombesin (BN) as a targeting peptide	^68^Ga^3+^	radiometal − chelator complexes	coprecipitation method	PET-MRI	Hajiramezanali et al. (2019)
carboxymethylcellulose (CMC)	just pH sensitive	^68^Ga^3+^	simultaneous cross-linking of CMC into NP and ^68^Ga^3+^ loading (direct)	lyophilization	PET	Piras et al. (2019)
chitosan and PGA biopolymers by self-assembly	–	^68^Ga^3+^	chelator-modified chitosan and ^68^Ga^3+^ loading (direct)	lyophilization	PET-MRI	Körhegyi et al. (2019)
core − shell NP of poly(2-oxazoline) block with 4-(bromomethyl) phenyl)-di-tert-butylfluorosilane	–	^18^F	radiometal − chelator complexes (irreversible covalent bonding)	microemulsion polymerization/ lyophilization	PET	Berke et al. (2018)
dendritic based polymer	melanin-targeting NP	^177^Lu	1-Ethyl-3-(3-dimethylaminopropyl )carbodiimide-mediated peptide coupling	thermal decomposition and functionalized with PEGylated PAMAM (Poly(amidoamine)) Dendron	MRI	Bordeianu et al. (2018)
polyvinyl pyrrolidone (PVP) as a surface capping agent for stabilizing the NP formed by ferric ion and gallic acid	–	^125^I	coordination reactions	coordination reactions between Fe3+ ions and gallic acid	SPECT-CT	Chen et al. (2018)

### 4.1. Advantages of radioactive polymeric nanoparticles

Rossin and coworkers (2005) developed polymeric NPs conjugated or not with folate and labeled with ^64^Cu. They observed that NPs with folate presented the same biodistribution results than NPs without folate. Furthermore, these NPs can accumulate in solid tumor, which is an interesting characteristic aiming cancer treatment (Rossin et al., 2005). Hamoudeh and colleagues (2007) prepared a PLLA NPs loaded with dirhenium decacarbonyl (Re_2_(CO)10^10^) and investigated the possibility of use this system for radionuclide intra-tumoral injection. They characterized the NPs containing Re and blank NPs and observed by an estimation using Mirdose 3.1 software that NPs loaded with (Re_2_(CO)10^10^) can deliver a high dose into a brain tumor and can be a promising intra-tumoral therapy (Hamoudeh et al., 2007).

There are some data that compare the radioactive NPs with free drug aiming tumor targeting. Lammers and coworkers (2008) developed two different systems using two different anticancer drugs. The first system was based on polymeric NPs containing doxorubicin and labeled with gadolinium containing or not immunoglobulin G. The therapeutic effect was evaluated and compared to free doxorubicin (Lammers et al., 2008). The other system was NPs containing gemcitabine uncleavable and cleavable and these particles were evaluated by therapeutic effect comparing with free gemcitabine. It was observed that the drugs encapsulated in polymeric NPs presented an increase of efficacy and reduction of toxicity in comparison with free drugs. Yadav and colleagues (2010) developed two different polymeric NPs (PLGA-MPEG and PLGA-Pluronic) containing etoposide labeled with ^99m^Tc and evaluated the blood clearance and biodistribution. It was verified that the two systems prepared showed higher concentrations in the circulation compared to free drug labeled with ^99 m^ (Yadav et al., 2010). Also, NPs produced by PLGA-MPEG presented the highest level in the blood up to 24 h. In biodistribution studies, it was detected that NPs were less uptake by spleen and liver in comparison with free etoposide. However, NPs had an increase of uptake in brain and bone, while free drug presented low levels of uptake in these parts. These results suggested that these NPs can be used as an interesting option for leukemia treatment. Ozgur and colleagues (2012) investigated the radiopharmaceutical potential of pheophorbide-a bovine serum albumin NPs labeled with ^99m^Tc. It was verified that NPs showed a higher uptake in the breast and uterus than pheophorbide-a labeled with ^99m^Tc and can be an option to use in scintigraphic tumor imaging and drug delivery (Ozgur et al., 2012). Other studies focus comparing radioactive polymeric NPs and radioactive substance alone. Wang and coworkers (2015) designed three polymeric NPs (PDLA-CS, PEG-PLGA-PLL, PEG-PS/CaP) aiming to tumor treatment. These systems were labeled with ^18 ^F-SFB and compared with each other and with ^18 ^F-SFB alone. ^18 ^F-SFB showed heart, liver and bladder tissues distribution without accumulation in tumor tissue. ^18 ^F-SFB-PDLA-CS NPs presented a systemic distribution with accumulation in tumor tissue, liver and bladder. The other NPs (^18 ^F-SFB-PEG-PLGA-PLL, ^18 ^F-SFB-PEG-PS/CaP) showed faster accumulation in tumor tissues than NPs prepared only PDLA-CS. According to these results, it can be suggested that NPs can be suitable for tumor treatment (Wang et al., 2015). Encapsulated Ru(phen)_2_(tpphz)^+2^ in PLGA nanoparticles labeled with ^111^In hEGF to oesophaegal cancer that overexpress EGRF treatment. It was verified that ^111^In-hEGF-PLGA NPs accumulated primarily in cytosol and presented greater level of internalized radioactivity than ^111^In-DTPA-hEGF peptide. Moreover, ^111^In-hEGF-PLGA-Ru1 NPs showed higher cytotoxic effect than ^111^In-hEGF-PLGA NPs. Also, ^111^InCl_3_ (free radioactive agent) had no impact in cell proliferation. However, ^111^In-hEGF-PLGA-Ru1 NPs had similar DNA damage than ^111^In-hEGF-PLGA NPs and hEGF-PLGA-Ru1 NPs. Furthermore, ^111^In-hEGF-PLGA NPs and ^111^In-hEGF-PLGA-Ru1 NPs presented similar uptake of radioactivity into the liver, spleen and kidney. These results suggested that ^111^In-hEGF-PLGA-Ru1 NPs can be an interesting option for EGFR-overexpressed esophageal cancer treatment (Gill et al., 2018). Gibbens-Bandala and colleagues (2019) investigated the breast cancer therapy using ^177^Lu-BN-PLGA NPs and ^177^Lu-BN-PLGA NPs containing paclitaxel (PTX). According to the in vivo studies, they observed that ^177^Lu-BN-PLGA-PTX NPs showed the lowest tumor proliferation, followed by PLGA-PTX NPs and ^177^Lu-BN-PLGA NPs. Also, ^177^Lu-BN-PLGA-PTX NPs presented the lowest metabolic tumoral activity, indicating that the combination of drug, polymer and radioactive agent increase the therapeutical response (Gibbens-Bandala et al., 2019).

Trujillo-Nolasco and coworkers (2019) prepared a hyaluronic acid PLGA NPs containing methotrexate (MTX) labeled with ^177^Lu for rheumatic arthritis local treatment and investigated the in vitro cellular uptake and cell viability. They observed that ^177^Lu-DOTA-PLGA-MTX NPs showed a passive nonspecific uptake, while ^177^Lu-DOTA-HA-PLGA NPs with or without MTX had an increase of uptake due to HA presence. Free MTX presented a cells inhibition of 50% at 48 h, while MTX encapsulated in PLGA NPs or in HA-PLGA NPs had an inhibition of 31% and 20% at 48 h, respectively. When the particles were labeled with ^177^Lu, the cytotoxic arrived 95% at 120 h. The ^177^Lu-DOTA-HA-PLGA-MTX NPs presented the highest inhibition compared to ^177^Lu-DOTA-HA-PLGA NPs and PLGA-MTX NPs. According to these results, ^177^Lu-DOTA-HA-PLGA-MTX NPs are a promising agent to treat rheumatic arthritis (Trujillo-Nolasco et al., 2019). Another study evaluated the biodistribution of hydroxyapatite (HA) PHPMA-TT NPs radiolabeled (Lobaz et al., 2019). They compared the biodistribution of ^99m^Tc-HEDP with ^99m^Tc-HEDP HAP NPs. It was observed that ^99m^Tc-HEDP accumulated in the liver and bones, while ^99m^Tc-HEDP HAP NPs accumulated in the liver and spleen. Besides, HAP NPs labeled in vitro or in vivo with ^99m^Tc-HEDP had same distribution profile (Lobaz et al., 2019).

## 5. Radioactive polymeric nanoparticles for biomedical applications

Figure 4 shows the possible systems of polymeric nanoparticles and examples are described in this section. They can be used for therapy and diagnostics.

Polymeric nanoparticulate systems are reported as excellent diagnostic, therapeutic, and theranostic precursor agents. These systems are used for studies in numerous diseases such as ischemia, cardiovascular diseases (Hwang et al., 2014), angiogenesis (Almutairi et al., 2009), atherosclerosis (Delgado et al., 2000; Subramanian et al., 2010; Stendahl and Sinusas, 2015; Wang et al., 2017), inflammation, cancer (Delgado et al., 2000; Subramanian et al., 2010; Criscione et al., 2011; Liu et al., 2011; Wang et al., 2017), and infectious diseases (Delgado et al., 2000; Subramanian et al., 2010; Criscione et al., 2011; Liu et al., 2011; Seo et al., 2014; Aweda et al., 2015; Fairclough et al., 2016; Woodard et al., 2016; Dos Santos et al., 2017; Tu et al., 2018; Simonetti et al., 2019), among others. The radiolabeled NPs decorated or functionalized with amino acids, simple peptides, or dendrimers promote targeting to improve uptake, biocompatibility, and stability. Low toxicity has been reported in different cells lines. Biodistribution shows elimination of particles in feces and urine with low retention into other tissues. However, a low profile of retentions by the monophagocyte system (liver and spleen) and prominent renal uptake (Almutairi et al., 2009; Hwang et al., 2014) were observed in some studies. This may be related to the properties of the nanoparticles which promote higher accumulation into these tissues (Delgado et al., 2000; Subramanian et al., 2010; Stendahl and Sinusas, 2015; Wang et al., 2017).

### 5.1. Cardiovascular diseases

Cardiovascular diseases are the leading cause of death worldwide. According to the World Health Organization (WHO), cardiovascular diseases are responsible for over 17.9 million deaths. In order to reduce the risk and increase prevention in this filed, initiatives on new therapeutics and diagnostics are essential (Cheng et al., 2017). Among all cardiovascular diseases, cardiac ischemia is one of the most prevalent. It occurs when blood flow is reduced, preventing the heart muscle from receiving enough oxygen, reducing the heart muscle’s ability to pump blood, leading to a heart attack. For early detection of the first signs of cardiovascular ischemia, Hwang and colleagues proposed the use of chitosan hydrogel loaded with vascular endothelial growth factor (VEGF) peptides and radiolabeled with ^99m^Tc administered in rats via apical puncture. The results demonstrated the efficacy of this nanosystem by decreasing the perfusion defect and increasing vascular density (Hwang et al., 2014).

In another example, Almutairi et al. assessed the angiogenesis process (responsible for cardiovascular ischemia) using a positron-emitting dendritic biodegradable nanoprobe (12 nm; heterobifunctional dendritic core chemoselectively functionalized with heterobifunctional polyethylene oxide chains that form a protective shell). The dendritic nanoprobe was targeted to αvβ3 integrin, a known marker of angiogenesis. In order to increase selectivity for integrins, peptides of cyclic arginine-glycine-aspartic acid (RGD) were added to the structure of dendritic nanoprobe. Each branch of the dendritic core was labeled with ^76^Br. In vivo studies in mice showed no specific organ accumulation and the nanosystems were cleared efficiently. In addition, these nanosystems showed a high accumulation of these NPs in angiogenic muscles, allowing them to collect highly selective images of the process of angiogenesis (Almutairi et al., 2009).

In a multi-modal blood pool imaging approach, Criscione and collaborators developed the ^99m^Tc-labeled G4-([[Ac)-DTPA]-mPEG_12_] dendrimer as a contrast agent in a micro SPECT/CT (Computed Tomography) hybrid imaging system in mice. Pharmacokinetics and biodistribution have shown that long intravascular residence time and almost exclusively renal clearance of the dendrimers provide useful tools for defining vascular and cardiac structures in the hybrid imaging system (PET-CT) (Delgado et al., 2000; Almutairi et al., 2009; Subramanian et al., 2010; Criscione et al., 2011; Stendahl and Sinusas, 2015; Wang et al., 2017). Liu and colleagues prepared a nanoprobe conjugated with DOTA (1,4,7,10-tetraazacyclododecane-1,4,7,10-tetraacetic acid)-C-type atrial natriuretic factor labeled with ^64^Cu and evaluated its performance for PET-CT imaging of NPR-C (natriuretic peptide clearance receptor) receptors in a mouse model with ischemia. This multivalent nanoprobe improved blood retention and increased selectivity showing high potential to evaluate other animal cardiovascular disease models (Liu et al., 2011). Lastly, in order to better understand atherosclerosis, which is a chronic inflammatory vascular disease related to high risk of myocardial infarction and cerebrovascular events, use of ApoE-/-, ^64^Cu-NP (LyP-1) 4-dendrimer-BAT (BAT((p-(bromoacetamido)benzyl)-1,4,8,11-tetraazacyclotetradecane-N,N″,N‴,N⁗-tetraacetic acid) in mice showed high uptake in the aortic root and descending aorta by PET/CT (Delgado et al., 2000; Subramanian et al., 2010; Criscione et al., 2011; Liu et al., 2011; Seo et al., 2014; Woodard et al., 2016). Woordard and colleagues analyzed NPs conjugated with C-type atrial natriuretic peptide in different amounts labeled with ^64^Cu to investigate potential atherosclerosis detection by PET imaging. They observed that NPs containing higher amounts of peptide (25%) demonstrated the highest specificity for Apo E-/-, suggesting a potential use for detection of atherosclerosis status and progression (Delgado et al., 2000; Subramanian et al., 2010; Criscione et al., 2011; Liu et al., 2011; Seo et al., 2014; Woodard et al., 2016). In addition, Stendahl and Sinusas described several options of radiolabeled nanoprobes for PET-SPECT imaging to investigate, in vivo, cardiovascular disease as ischemia, angiogenesis, or atherosclerosis (Delgado et al., 2000; Subramanian et al., 2010; Stendahl and Sinusas, 2015; Wang et al., 2017).

Another study reported the multimodality molecular nanoprobe imaging that can be used for clinical use in order to investigate cardiovascular diseases. There are some nanoprobes developed to evaluate atherosclerosis, such as dextranated-DTPA-modified magneto-fluorescent nanoparticles labeled with ^64^Cu that show accumulation in the aortic root and arch of atherosclerotic arteries of Apo E-/-, and CLIO (cross-linked iron oxide)-Cy5.5 iron oxide nanoparticle linked a peptide sequence that binds VCAM-1 (Vascular cell adhesion protein 1), which is a biomarker for atherosclerosis. In order to investigate thrombus formation, a fibrin-targeted PET probe attached to DOTA and labeled with ^64^Cu showed interesting results. Moreover, a folate-conjugated porphyrin nanoparticle labeled with ^64^Cu is multimodal molecular imaging proposed to evaluate post-myocardial infarction (Criscione et al., 2011; Liu et al., 2011; Seo et al., 2014; Fairclough et al., 2016; Woodard et al., 2016; Tu et al., 2018).

### 5.2. Infectious diseases

Infectious diseases can be caused by a pathogen, such as viruses, bacteria, fungi, and parasites. These microorganisms multiply rapidly and alter homeostasis. The prevalence of diseases such as viral hepatitis, cholera, malaria, dengue fever, human immunodeficiency virus (HIV), Ebola, salmonella, influenza, and severe respiratory syndrome is closely associated with an increase in the totality and morbidity index. Thus, the development of new technologies for the diagnosis and therapy of these manifestations is necessary (Lim et al., 2016; Wang et al., 2017).

Fairclough et al. aimed to improve the diagnosis of the inflammatory process as the inflammation foci when using radiolabeled chitosan-leukocytes. The radionuclides used in the NPs labeling process were ^89^Zr and ^64^Cu. The results showed that the ^89^Zr-chitosan NPs showed a lower efflux than the ^64^Cu-chitosan NPs. In addition, the high reproducibility of the methodology and leukocyte monitoring made it possible to monitor inflammatory and infectious foci (Fairclough et al., 2016). Aweda and colleagues investigated pharmacokinetics and biodistribution of two systems (silver-loaded polyphosphoester NPs and N-heterocyclic silver carbene complex) labeled with ^111^Ag for the antimicrobial effect of silver ions. These NPs were administered by nebulization and showed good retention in lungs. Thereby, it was verified that the nanosystems using silver for therapeutic applications labeled with ^111^Ag can be used as theranostics due to the presence of ^111^Ag which evaluates the pharmacokinetics and biodistribution of silver nanosystems (Aweda et al., 2015).

Simonetti and colleagues evaluated pterostilbene or crude extract from non-fermented grape pomace loaded in poly(lactide(co-glycolide)) (PLGA) NPs with six coumarin fluorescent probes against *Candida albicans* biofilm. A significant inhibition of *C. albicans* biofilm using these PLGA NPs was observed (Simonetti et al., 2019). Santos et al. evaluated betamethasone and dexamethasone PLA (Poly-lactic acid) NPs labeled with ^99m^Tc in *Staphylococcus aureus* infection/inflammation in vivo model. They verified that ^99m^Tc-PLA NPs containing betamethasone showed accumulation at *S. aureus* inflammation site indicating that this system can be used for infection/inflammation foci during in vivo detection (Dos Santos et al., 2017). Another example from Helal-Neto et al. described the development of an ethambutol NP using PCL (poly-caprolactone) as a polymer and radiolabeled with ^99m^Tc. The results showed that this NP had an in vitro and in vivo theragnostic effect in *Mycobacterium bovis* strain (Helal-Neto et al., 2019).

### 5.3. Cancer

Cancer is the second leading cause of death in the world. In 2018, cancer was responsible for approximately 9 million deaths. Due to this, the development of new diagnostic and therapy technologies is essential for improving the population’s quality of life. In this direction, many radio-NPs have been developed for diagnosis and/or therapy, such as ^99m^Tc-PLGA-NPs for lung cancer imaging (Delgado et al., 2000; Piras et al., 2019) or sentinel lymph nodes (Subramanian et al., 2010).

Delgado and collaborators developed PLGA and PEG-PLGA microspheres radiolabeled with ^99m^Tc for lung perfusion imaging and therapy and compared them in vivo biodistribution with ^99m^Tc-HAM (albumin microspheres) as a reference. The authors observed that only ^99m^TC-PLGA and ^99m^Tc-PEG-PLGA microspheres prepared with Poloxamer 188 as a stabilizer showed similar accumulation to ^99m^Tc-HAM in the lung. Other stabilizers presented preferential accumulation in the liver (Delgado et al., 2000). Subramanian and colleagues developed ^99m^Tc-PLGA NPs and evaluated their biodistribution and scintigraphic imaging as an alternative to ^99m^Tc-sulfur colloid/albumin colloid for sentinel lymph node detection. Scintigraphic images showed ^99m^Tc-PLGA NPs in the sentinel node. Moreover, these NPs accumulated in the popliteal and iliac nodes for 3 h. Thus, these NPs can be an exciting option for sentinel lymph node detection. However, more studies need to be done to confirm these results (Subramanian et al., 2010). Another study evaluated etoposide PLGA-MPEG (Poly(ethylene glycol) methyl ether-block-poly(lactide-co-glycolide) and etoposide PLGA-Pluronic NPs labeled with ^99m^Tc for leukemia therapy (Yadav et al., 2010). The in vitro release profile and in vivo biodistribution of these NPs were investigated. The in vitro release profile of drugs encapsulated in PLGA-MPEG and PLGA-Pluronic NPs showed slower release (26.6% and 45.6% in 12 h, respectively) than free etoposide, which completely released in 4 h. Furthermore, free drug presented accumulation in lung, liver, and spleen, while etoposide PLGA-MPEG and PLGA-Pluronic NPs labeled with ^99m^Tc had higher concentrations in the blood. These results suggest that these NPs can improve the treatment of leukemia using etoposide (Yadav et al., 2010). Banerjee and colleagues developed ^111^In-PSMA(Prostate-specific membrane antigen)-targeted PLA-based NPs radiolabeled for SPECT imaging of PSMA-expressing tissues (Banerjee et al., 2017). The authors investigated the pharmacokinetics and biodistribution of these NPs. In addition, they compared the results for targeted and untargeted NPs. The results showed a similar accumulation of ^111^In-PSMA-decorated NPs and untargeted NPs. Nonetheless, this study provided some information about radiolabeled particles, which can be used for predicting tumor accumulation in the human body (Banerjee et al., 2017).

Another delivery system developed by He and collaborators for clinical application purposes used K237 polypeptide and folic acid in combination with PLGA-PEG NPs. The biodistribution and pharmacokinetics were determined by radiolabeling the NPs with ^99m^Tc. The results showed tumor uptake in addition to other organs such as liver, kidney, and bladder. As a result, these developed NPs showed great potential for use, especially for tumors expressing VEGF receptor-2 and folate receptor, targets of K237 polypeptide and folic acid, respectively (He et al., 2016). Santos do Carmo et al. developed PLGA NPs loaded with anti-mucin-1 (MUC1) aptamer and radiolabeled with ^99m^Tc. The results showed that the system was able to accumulate specifically in the tumor, generating an excellent SPECT image. Furthermore, the authors suggested that an alteration of the ^99m^Tc radionuclide by a β (177-Luthetium) or α emitter (223 Radium) could promote a therapeutic effect on the tumor (Carmo et al., 2017). In order to develop a theragnostic nanostructure for esophageal cancer, Gill et al. developed PLGA NPs decorated with DTPA (diethylenetriaminepentaacetic acid) and hEGF (human epidermal growth factor) and loaded with the ruthenium (II) polypyridyl complex, such as Ru (phen) 2 (tpphz) 2+ (phen = 1,10-phenanthroline, tpphz = tetrapyrido(3,2-*a*:2′,3′-*c*:3′′,2′′-*h*:2′′′,3′′′-*j*)phenazine) also called Ru1, which is a radiosensitizing structure. This NP was radiolabeled with ^111^In. As a result, a significant reduction in cell survival occurred in EGRF high-expressing esophageal cancer cells in comparison with normal EGRF expression cells. The association of the Auger electron emitted by ^111^In and the radiosensitizing capacity of Ru1 was essential for increasing DNA damage to these cells (Gill et al., 2018).

Oda and colleagues produced polymeric micelles functionalized with DTPA and labeled with ^99m^Tc and evaluated in vivo biodistribution and scintigraphic images of this system. They observed high uptake in the liver, spleen, and kidney. However, polymeric micelles showed more selectivity for tumor targeting, which suggested that this system can be interesting for drug delivery and diagnostic monitoring, simultaneously (Oda et al., 2017). Another study evaluated the theranostic effect of core-shell silver NPs, with or without polyvinyl pyrrolidone (PVP), labeled with ^125^I and containing (or not) doxorubicin. It was verified that silver polymeric NPs containing doxorubicin radiolabeled with ^125^I presented the highest uptake in the tumor site. This system presented promising results for theranostic use for solid tumor treatment (Farrag et al., 2017). Berke and colleagues developed four polymeric core-shell nanoparticles functionalized with an organosilic fluoride acceptor, labeled with ^18 ^F and investigated by PET images for NP tumor uptake and clearance. These particles presented good tumor uptake and indicate an interesting option for theranostic effects (Berke et al., 2018). Liang and colleagues prepared an oligomeric NP conjugated with a folic acid receptor and radiolabeled with ^99m^Tc. These NPs presented selectivity for folate receptor in tumor cells and due to their selectivity, can be an option for SPECT/CT imaging (Liang et al., 2018).

Another example for a possible theranostics system is the development of nanoparticles conjugated with arginine-glycine-aspartic acid peptide, polyethylene glycol, and croconaine dye labeled with ^125^I. These NPs showed preference for angiogenic tumor vessels favoring their possible theranostic use (Tang et al., 2018). Gibbens-Bandala and colleagues evaluated a paclitaxel PLGA NP conjugated with bombesin (gastrin-releasing peptide that binds to gastrin receptors overexpressed in breast cancer) labeled with ^177^Lu. They observed a controlled release of paclitaxel as well as a higher uptake of tumor cells suggesting an interesting system with therapeutic and diagnostic effects (Gibbens-Bandala et al., 2019). Another system described for breast cancer treatment and detection was based on magnetic NPs coated with chitosan, conjugated with a radioisotope chelator and bombesin labeled with ^68^Ga. It was verified that bombesin conjugated with NPs can be an interesting binder for gastrin receptor of tumor cells. Moreover, these NPs can be used in PET/MRI imaging for detection of breast, lung, and prostate cancers (Hajiramezanali et al., 2019).

## 6. Clinical application and trial

Although a great variety of radioactive nanoparticles have reached the pre-clinical status, just a few have definitively reached clinical/commercialization phase and all of them as non-polymeric nanoparticles. Most of them are: colloidal nanoparticles, liposomes and gold-nano-metal nanoparticles.

The first case is the 99mTc-colloid (99mTc-Nanocoll^®^) a nano-colloid albumin deserve attention. This radioactive nanodrug has been approved for lymph nodes imaging in EU (Gommans et al., 2001; Mariani et al., 2001; Gommans et al., 2009) and a modified version of the original 99mTc-Nanocoll using sulfur in the colloidal state (99mTc-Technecoll^®^) has been approved in USA for nodal detection in melanoma, prostate and breast cancer (Alazraki et al., 1997; Holl et al., 2009; Seok et al., 2010; Ganswindt et al., 2011; Seo et al., 2011; Zhang et al., 2019). Back in the 80-90’s was developed and clinically evaluated an 111In-encapsulating liposome formulation (Vescan) for detection of carcinoma and metastases of prostate, lung and breast cancer (Presant et al., 1994; Jensen and Bunch, 2007). However, due the lack of precision in detection rate of known tumors and improved alternate methods for tumor detection lead to the end of the program. (Jensen and Hodgson, 2020). The 99mTc-SnF2 registered under the name of Hepatate^®^ is a nanoparticle formed of Stannous fluoride colloid radiolabeled with technetium 99 m clinically used for lymphoscintigraphy: gastrointestinal, liver and spleen (Hirsch et al., 1989; Thakor et al., 2016). Finally, radioactive gold nanoparticles have shown to be an excellent radiosensitizer for great variety of cancer cells (Xu et al., 2020; Xuan et al., 2020). Regarding clinical trial, there was a phase I clinical trial conducted by Katti et al, on nanoparticle for thermotherapy (NCT00848042) (Thakor et al., 2016).

Polymeric radioactive nanoparticles have showed to be promising for translational studies. Although it has showed to have high efficacy, it haven’t presented many commercial forms when compared to traditional radiopharmaceuticals. However, Health institutes as NIH and NCI have been founding diverse clinical trials to study radioactive nanoparticles, including polymerics, for image and therapeutic uses (Zakeri et al., 2019). In 2017, FDA Approves Novel Radio-peptide Targeted Therapy Clinical Trial for Neuroendocrine Cancer (Hennrich and Kopka, 2019). Lutathera^®^ combines the radionuclide 177Lu with the somatostatin analogue DOTA-TATE to deliver ionizing radiation specifically to tumor cells expressing somatostatin receptors. desferrioxamine-based BFCAs for 89Zr have been reported with improved stability that permits reliable in vivo evaluation of polymeric materials (Deri et al., 2014; Pant et al., 2017). Several studies have investigated radioactive-iodine-labeled functional nanomaterials for cancer treatment. Liu et al. used albumin nanoparticles containing paclitaxel (PTX), a potent chemotherapeutic drug. This material showed prolonged blood circulation time, specific tumor uptake, and high intratumor penetration ability. The combined therapeutic effects (chemo- and radiotherapy) of 131I-HSA-PTX were found to be highly effective in the 4T1 cancer xenograft model compared to radiotherapy- and chemotherapy-alone groups (Tian et al., 2017).

## 7. Future aspects, challenges, and applications

The future of medicine relies on the ability of two main pillars: new drugs and new equipments. In this direction the use of radioactive polymeric nanoparticles that are able to simultaneously treat and diagnose is quite an advancement in the medicine field. In addition, the use of highly specific radioactive polymeric nanoparticles, which have been developed for targeting one, and just one, tissue/organ reduces the amount of radioactive material used, thus reducing the risk of the use of radiation in human body. Regarding challenges, it is important to note that technical apparatuses and methodological approaches which improve synthesis (scale up), biodegradability (body clearance with the minimum environmental impact), size, shape, surface charge, and surface modification (in order to increase exponentially the affinity for a specific tissue/organ) are still required. Nonetheless, the safe use of the final product must be established; in this direction a well-defined protocol must be provided, pointing to which specific assays must be performed and which parameters are considered safe for human use, especially in the case of radioactive polymeric nanoparticles, which already have a radiation source included (as radiopharmaceuticals) and demand more careful analysis of each parameter. Despite these challenges, a great field of application is open. Radioactive polymeric nanoparticles represent the future of nuclear medicine in both aspects: diagnosis and therapy. In the field of diagnostics, the use of radioactive polymeric nanoparticles may reduce the radiation dose used to perform a SPECT or PET-CT scan as the time necessary to perform the exam, which is highly targeting. In terms of therapy (molecular radiotherapy), the use of radioactive polymeric nanoparticles may reduce the adverse effect of injury in the surrounding tissues (healthy tissues) and increase treatment efficacy.

## 8. Conclusions

New emerging radiolabeled polymeric nanoparticles are revolutionizing medicine in terms of diagnostics, treatment, and theranostics. These radionuclides include polymeric NPs, liposomal carriers, dendrimers, magnetic iron oxide NPs, silica NPs, carbon nanotubes, and inorganic metal-based nanoformulations. Between these nano-platforms, polymeric NPs are of great interest in the biomedical field due to their excellent properties such as surface to mass ratio, quantum properties, biodegradability, low toxicity, and ability to adsorb and carry other molecules. In addition, these NPs are capable of carrying high payloads of radionuclides and/or drugs and can be used for diagnostic, treatment, and as theranostics depending on the radioactive material used.

Despite advancements in the development of radiolabeled NPs, there are still several facts which require special attention, namely synthesis routes and the toxic effects of these nanomaterials.

Through the development of radiolabeling polymeric nanoparticles with multi-modality properties, concerns about their safety have been raised. Like any radioactive modality, concerns about risks are always relevant but data in the literature corroborate that the benefits of using radioactive material outweighs its risks (Kunjachan et al., 2015; Simonetti et al., 2019). Similarly, imaging modalities like CT procedures that may induce DNA damage are used when the benefits outweigh the risks (Dos Santos et al., 2017; Helal-Neto et al., 2019; Basheerudeen et al., 2017; International Society for Pharmacoeconomics and Outcomes Research Rare Disease Special Interest Group, 2015). The use of radioactive polymeric nanoparticles may also improve efficacy, leading to a higher targeting in tissues/organs (Bolzati et al., 2012; Banerjee et al., 2017), which decreases the dose used (including the radioactive dose) and consequently reduces the risk. Moreover, Voigt et al. (Voigt et al., 2014; He et al., 2016) reported that several polymeric nanoparticles were nontoxic. On the other hand, Grabowski et al. (Grabowsk et al., 2015; Carmo et al., 2017) demonstrated that polymeric nanoparticles using the same polymer (PLGA), but produced by a different method, may show distinct profiles of cytotoxicity. This kind of information demonstrates that there is no consensus in the field and a more appropriate and definitive assessment must be provided.

The use of radioactive polymeric nanoparticle may represent the future of nuclear medicine in both diagnosis/imaging and therapy or both properties may be gathered into a single drug like in theranostics. The methodologies to produce radioactive nanoparticles are reliable and allow for scale-up. Nonetheless, the polymeric nanoparticles themselves can be produced as lyophilized kits to be radiolabeled in a hospital and/or centralized radiopharmacy and used for patients in an in-house production system.

Among the advantages claimed by the radioactive polymeric nanoparticles, the higher targeting for a specific site is one of the best, because it may represent a lower dose of radioactive material in the final drug while producing the same effect. Furthermore, higher targeting may represent an early diagnosis which means more time to treat the patient with the disease in a more sensitive phase.

Finally, radioactive polymeric nanoparticles represent an evolution. These applications combined with the safety of these nanosystems may represent the future of medicine.
